# *Salvia miltiorrhiza* Bunge (Danshen) for Treatment and Prevention of Urolithiasis: A* Drosophila* Animal Study

**DOI:** 10.1155/2019/1408979

**Published:** 2019-01-20

**Authors:** Wen-Chi Chen, Tzu-Yang Chou, Huey-Yi Chen, You-Rong Yang, Kee-Ming Man, Ming-Yen Tsai, Yung-Hsiang Chen

**Affiliations:** ^1^Graduate Institute of Integrated Medicine, College of Chinese Medicine, China Medical University, Taichung, Taiwan; ^2^Departments of Urology, Obstetrics and Gynecology, and Medical Research, China Medical University Hospital, Taichung, Taiwan; ^3^Department of Chinese Medicine, Kaohsiung Municipal Gangshan Hospital, Kaohsiung, Taiwan; ^4^Department of Medicinal Botanicals and Health Applications, Da Yeh University, Changhua, Taiwan; ^5^Department of Anesthesiology, China Medical University Hsinchu Hospital, Hsinchu, Taiwan; ^6^Department of Chinese Medicine, Kaohsiung Chang Gung Memorial Hospital and Chang Gung University College of Medicine, Kaohsiung, Taiwan; ^7^Department of Psychology, College of Medical and Health Science, Asia University, Taichung, Taiwan

## Abstract

Traditional Chinese medicine (TCM) has been prescribed for the treatment of stone disease for thousands of years.* Salvia miltiorrhiza* (Danshen) was previously shown to have potential for treatment of stone disease in animal and clinical studies. In this study, we further studied the antiurolithiasis effect of Danshen in a fly model. Wild-type male* Drosophila melanogaster* CS flies were used in this study, with 0.25% ethylene glycol (EG) as a lithogenic agent. 2% potassium citrate (K-citrate) was the positive control agent for prevention (all agents added at the start of experiment) and treatment (drugs added after 2-week addition of lithogenic agent) studies compared with 15, 30, and 60 *μ*g/ml of Danshen extract. In the prevention study, both 2% K-citrate and Danshen (30 and 60 *μ*g/ml) significantly inhibited EG-induced calcium oxalate (CaOx) crystal formation. In the treatment study, only 2% K-citrate and high-dose of Danshen (60 *μ*g/ml) significantly inhibited EG-induced CaOx crystal formation. Survival analysis for EG with Danshen was compared with that for EG with K-citrate. The mean lifespan was significantly reduced by administration of EG, and the results in the Danshen group were similar to those in the control group. In conclusion, Danshen revealed both preventive and treatment effects on CaOx crystal formation in a fly model.

## 1. Introduction

Kidney stones are common, with an increasing worldwide incidence and prevalence, irrespective of sex, race, and age [1, 2]. Dietary habits can be a contributing factor [3]. However, long-term change in dietary habits is difficult to accomplish. Oral potassium citrate (K-citrate) is an effective preventive agent but should be taken daily. Patient noncompliance and side effects may interfere with efficacy. Therefore, more effective or better-tolerated drugs are needed.

Traditional herbal medicine has long been used for stone disease [4]. Our prior study focused on* Salvia miltiorrhiza* Bunge (Danshen), an herbal drug used in traditional Chinese medicine (TCM). Danshen has been prescribed in Taiwan for many diseases. We used the database of the Taiwan National Health Insurance to study the potential clinical effect of TCM herbs on urolithiasis [1]. The study found that Danshen use was associated with a decreased rate of stone treatment and that long-term Danshen use was not associated with increased bleeding risk. Another screening study of medicinal herbs for use in prevention of urolithiasis in an animal model also found that Danshen effectively decreased the rate of calcium oxalate (CaOx) crystal formation [1]. Therefore, we plan to examine both preventive and treatment effects of Danshen in additional animal studies and to assess tolerability by comparing survival in treatment and control groups. The results may suggest that this classic Huoxue Huayu herb (a TCM term used to describe an agent that promotes blood circulation, relieves pain, and treats blood stasis) can be further applied in a human clinical trial.

## 2. Material and Methods

### 2.1. Preparation of Lithogenic Flies and Stock

In this study, animal for the lithogenesis study was wild-type male* Drosophila melanogaster* CS flies. The preparation of experiment method was according to our previous published studies [4, 5]. In brief, flies were breed in plastic vials containing standard medium for fly (agar, yeast, corn syrup, and sugar), at 25°C, 50–60% humidity, with a 12-h light–dark cycle. The vials were changed twice a week. The 0.25% ethylene glycol (EG) lithogenic and treatment agents were added in the above standard medium as needed.

### 2.2. Prevention and Treatment of Danshen on Fly CaOx Crystal Formation

This study of fly CaOx crystal formation was divided into two experimental models, prevention and treatment. The lithogenic agent EG (0.25%) (wt/vol) was added in the fly medium in each group of flies. The first experiment was designed as comparative preventive effect of 2% potassium citrate (K-citrate, serving as positive control) and 15, 30, and 60 (*μ*g/ml) Danshen. All the agents were added since the start of experiment until the end of study.

The other experiment was designed as treatment effect of 2% K-citrate and 15, 30, and 60 *μ*g/ml Danshen. Flies were feed with 0.25% EG from the beginning and last to the end of experiment. The addition of K-citrate and 15, 30, and 60 *μ*g/ml Danshen started from the third week to the end of experiment. After 3 weeks, the flies (200 flies for each group) were killed under CO_2_ narcotization, and the Malpighian tubules were removed under microscopy. Dissection and processing tubules were observed under polarized light microscopy (Olympus BX51 optical microscope, Tokyo, Japan).

### 2.3. Survival Analysis of* Salvia miltiorrhiza* Bunge, K-Citrate, and EG

The lifespan assay for Danshen, K-citrate, and EG groups was performed on a fly model according to our previous studies [4, 5]. In brief, new fly emergents were collected in foam plugs and kept horizontally. Flies were divided into four groups (n ≅ 150 in each group) in terms of control, 0.25% EG, 2% K-citrate, and Danshen (60 *μ*g/ml) groups. Survivors in each vial were counted and dead flies were removed daily. Lifespans of each group were compared to Danshen group and tested for significance with log-rank test.

### 2.4. Polarized Light Microscopy Observation

The relevant aspects were photographed and the scales were obtained. The degree of CaOx crystal formation in each group was recorded and calculated. The degree of CaOx crystal formation was defined as grade 1, 2, and 3 according to previous studies [4, 5]. The crystal formation (%) was calculated by total number of crystal formation.

### 2.5. Statistical Analyses

One-way analysis of variance (ANOVA) was applied to detect overall differences among the groups; for all multiple comparisons, Bonferroni correction was applied. Significantly different groups were compared pairwise using the Mann–Whitney U test for crystal scores. All statistics were done using the SigmaStat software (SPSS; Systat Software, San Jose, CA). The statistical analyses were set at* P* < 0.05 as statistical significance.

## 3. Results

**Figure 1:**
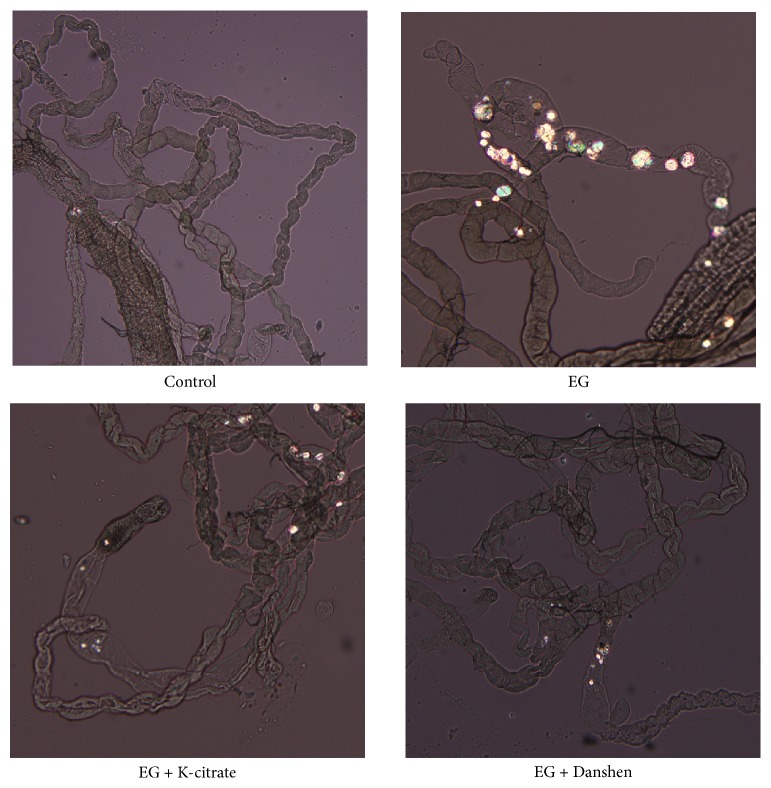
Effects of K-citrate and Danshen on EG-induced crystal deposition in the Malpighian tubules of* Drosophila*. The images show representative polarized microscopy for the flies with 0.25% EG-induced crystal formation in Malpighian tubules.

Compared with the control group, EG-induced crystal formation in* Drosophila* Malpighian tubules was clearly observed using microscopy (Figure 1) and was previously identified as CaOx [6]. Both K-citrate and Danshen effectively decreased the rate of CaOx crystal formation.

**Figure 2:**
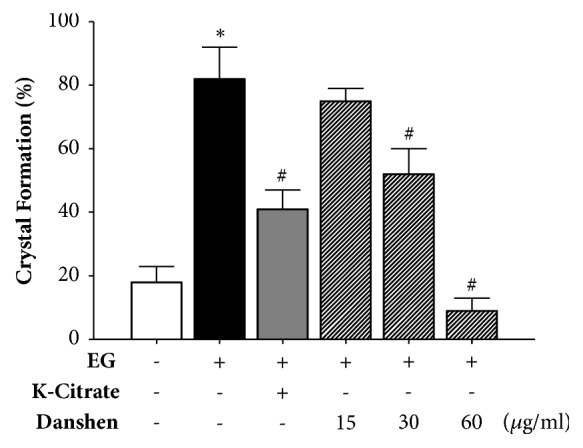
In the prevention study, the rates of CaOx crystal formation. Crystal formation in 0.25% EG, 2% K-citrate, and 15, 30, and 60 *μ*g/ml Danshen-treated* Drosophila* (n *≅* 150 for each group). *∗P* < 0.05, compared to the control. ^#^*P* < 0.05, compared to the 0.25% EG-treated group.

In the prevention study, after 21 days, the rates of CaOx crystal formation in the control, 0.25% EG, 2% K-citrate, and 15, 30, and 60 *μ*g/ml Danshen groups were 18.2%, 82.1%, 41.3%, 75.8%, 52.4%, and 9.5%, respectively. Both 2% K-citrate and Danshen (30 and 60 *μ*g/ml) significantly inhibited EG-induced CaOx crystal formation (Figure 2).

**Figure 3:**
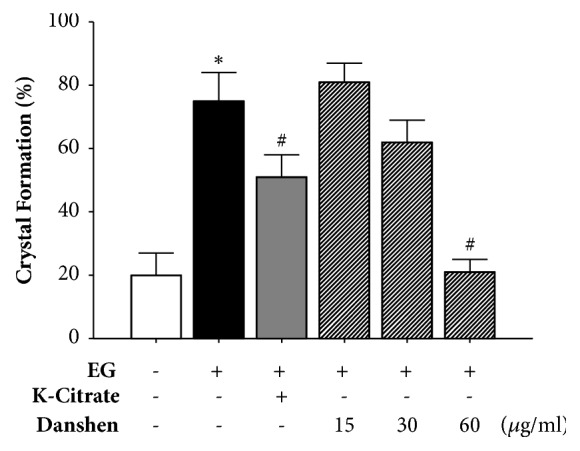
In the treatment study, the rates of CaOx crystal formation. Crystal formation in 0.25% EG, 2% K-citrate, and 15, 30, and 60 *μ*g/ml Danshen-treated* Drosophila* (n *≅* 150 for each group). *∗P* < 0.05, compared to the control. ^#^*P* < 0.05, compared to the 0.25% EG-treated group.

In the treatment study, the rates of CaOx crystal formation in the control, 0.25% EG, 2% K-citrate, and 15, 30, and 60 *μ*g/ml Danshen groups were 20.2%, 75.1%, 51.8%, 81.0%, 62.5%, and 21.4%, respectively. Only 2% K-citrate and high-dose of Danshen (60 *μ*g/ml) significantly inhibited EG-induced CaOx crystal formation (Figure 3).

**Figure 4:**
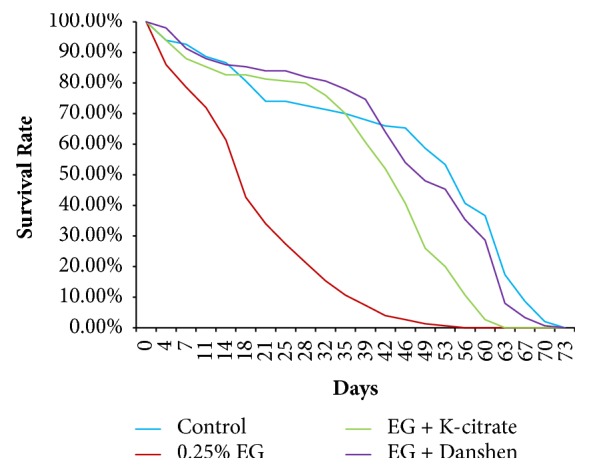
Lifespan of control, 0.25% EG, EG + 2% K-citrate, and EG + 60 *μ*g/ml Danshen-treated flies.

Survival analysis for EG with Danshen was compared with that for EG with K-citrate. This analysis was performed to determine the effect of Danshen with lithogenic agents on lifespan and mortality. The control flies had mean and maximum lifespans of 46.0 and 73 days, respectively. The mean lifespan was significantly reduced by administration of EG, with a mean of 24.1 days and maximum of 55 days. The results in the Danshen group were similar to those in the control group, with a mean of 45.3 days and maximum of 73 days (Figure 4).

## 4. Discussion

Danshen has both preventive and treatment effects on CaOx crystal formation in a lithogenic fly model. In the preventive study, high-dose Danshen (60 *μ*g/ml) was more effective than K-citrate. More interestingly, in the treatment study, the effect of drug treatment with Danshen was much greater than that of K-citrate. In clinic, K-citrate is used for prevention rather than treatment of urolithiasis. For existing CaOx crystals, K-citrate has less treatment benefit. Thus, the results were comparable to those reported by Chung et al. [7].

Danshen is safe and did not shorten the lifespan of flies in this study, even when combined with lithogenic EG. The average and maximum lifespan were similar to that of the control group. Although K-citrate can prevent crystal formation, both the mean and maximum lifespan were reduced by the combination with EG. The results revealed that long-term treatment with K-citrate for prevention of CaOx crystal formation in animals may possibly have some deleterious side effects with unknown cause. This result was compatible with that of our other studies using hydroxycitrate (a derivative of citric acid that is found in a variety of tropical plants including* Garcinia cambogia* and* Hibiscus sabdariffa*) and K-citrate for the prevention of CaOx crystal in flies (unpublished data).

Danshen was reported to have pharmacologic effects in coronary heart disease, including antioxidative, anti-inflammatory, and endothelial protective effects [7], as well as inhibition of atherosclerotic plaque formation and neointimal hyperplasia [8], reduction of myocardial oxygen consumption, improved energy metabolism, and protection of cardiomyocytes [9, 10]. Danshen also has an effect on blood vessels, through inhibition of platelet adhesion and aggregation [9, 10] and improvement of microcirculation [11]. This effect may imply a risk of bleeding tendency with use of Danshen. In our previous study, Danshen did not increase bleeding events in a national population-based study of clinical use [4]. Reported side effects of oral Danshen were occasional gastrointestinal discomfort, sensation of head fullness, and facial flushing [12]. Therefore, Danshen is safe for treatment of CaOx stone disease.

In TCM, Danshen is made of the dried root and has been clinically used for more than 2,000 years [13]. Although more than 20* Salvia* species are referred to as Danshen, the true Danshen is* S. miltiorrhiza* according to the Chinese Pharmacopoeia [14]. According to the Compendium of Materia Medica (Bencao Gangmu, Ming dynasty, 1596 AD), Danshen was commonly prescribed for treatment of blood circulatory disorders with the following functions: promotion of blood flow in menstruation, activation of blood circulation, removal of blood stasis, clearing of heart fire, relief of pain, resolving mental uneasiness and restlessness, and nourishing the blood [15]. Several reports have used Danshen to treat cardiovascular disease, osteoporosis, and cancer, and a hepatoprotective effect has been described [15, 16].

We have tested 80 herbs for the potential prevention of stone disease and Danshen was one of the effective agents [5]. Danshen showed an inhibitory effect on EG-induced CaOx formation in flies. The CaOx crystal formation in Malpighian tubules of* D. melanogaster* was significantly inhibited. Furthermore, we conducted a nationwide population study of clinical use of Danshen by stone patients and found that that its use was associated with a decreased rate of subsequent surgical treatment [1]. Therefore, we conducted this animal study to confirm the data. Danshen revealed its preventive effects for the crystal formation in a fruit fly. It is often used to treat cardiovascular diseases due to its efficacy on blood circulation. However, cardiac and renal dysfunctions often occur simultaneously due to the shared causes and pathogenesis [17, 18]. According to the epidemiological studies, urolithiasis is associated with various chronic diseases such as diabetes, metabolic syndrome, chronic kidney disease, hypertension, or cardiovascular diseases [19–23]. The correlation between these diseases and urolithiasis is most likely the result of a similar pathophysiological mechanisms.

In addition, oxidative stress is also considered as an important determinant of the common cause. Oxidative stress is the common feature between urolithiasis and venereal diseases [17, 24]. Further evidence showed that oxidative stress is also produced in idiopathic CaOx kidney stones. Thus, a kidney stone is not only a physical-chemical event but also a metabolic disorder.

An animal study had the advantage of a large experimental number, is economical, and yields quick results. Although Danshen had both preventive and treatment effects on CaOx crystal formation in this study, there were some limitations. Our study animal was an invertebrate without a true kidney. The study animal may be too simple to represent true kidney function in humans. Furthermore, the animal lifespan is too short to verify the long-term effect in humans [25–27]. Although the lifespan with use of K-citrate was shorted than in the control, there is a lack of clinical data on side effects in patients with CaOx stones.

## 5. Conclusion

Danshen has the potential for both treatment and prevention of CaOx crystal formation. Based on this animal study and previous clinical data, Danshen merits further clinical study to confirm its pharmacological effect in humans.

## Data Availability

The data used to support the findings of this study are available from the corresponding author upon request.
